# Dietary prebiotics promote intestinal *Prevotella* in association with a low-responding phenotype in a murine oxazolone-induced model of atopic dermatitis

**DOI:** 10.1038/s41598-020-78404-0

**Published:** 2020-12-03

**Authors:** Ann Laigaard, Lukasz Krych, Line F. Zachariassen, Lea Ellegaard-Jensen, Dennis S. Nielsen, Axel K. Hansen, Camilla H. F. Hansen

**Affiliations:** 1 Department of Veterinary and Animal Sciences, Faculty of Health and Medical Sciences, University of Copenhagen, Frederiksberg C, Denmark; 2 Department of Food Science, Faculty of Science, University of Copenhagen, Frederiksberg C, Denmark; 3 Department of Environmental Science, Aarhus University, Roskilde, Denmark; 4 Section of Experimental Animal Models, Department of Veterinary and Animal Sciences, Faculty of Health and Medical Sciences, University of Copenhagen, Ridebanevej 9, 1870 Frederiksberg C, Denmark

**Keywords:** Immunology, Allergy, Atopic dermatitis, Microbiota, Nutrition

## Abstract

Atopic dermatitis is a chronic eczema commonly observed among children in Western countries. The gut microbiota is a significant factor in the pathogenesis, and ways to promote intestinal colonizers with anti-inflammatory capabilities are therefore favorable. The present study addressed the effects of a prebiotic, xylooligosaccharide (XOS), on the gut microbiota and ear inflammation in an oxazolone-induced dermatitis model in BALB/c mice. Mice were fed a XOS supplemented or a control diet throughout the experiment. Ear thickness and clinical skin inflammation were scored blindly after three weeks topical challenge with 0.4% oxazolone. The mice were divided into high and low responders to oxazolone-induced dermatitis based on clinical inflammation and histological evaluation of ear biopsies, and significantly fewer high responders were present in the XOS fed group. In addition, XOS fed mice had higher abundance of *Prevotella* spp. in their gut microbiota compared to the control fed mice. Serum IgE and ear tissue cytokine levels correlated significantly with the clinical scores, and with the abundance of *Prevotella* spp. The strong association between the low-responding phenotype and high abundance of *Prevotella* spp., indicates an alleviating effect of this intestinal colonizer in allergic sensitization. *Prevotella* should be considered as a relevant target for future microbiota-directed treatment strategies in atopic patients.

## Introduction

Atopic dermatitis is a chronic intermittent and pruritic eczema with an infancy or early childhood onset, and it is a very common skin disease among children in Western countries^1,2^. The majority of atopic dermatitis patients (approx. 80%) develop an immunoglobulin E (IgE) associated form of dermatitis, with high serum IgE titers, formerly known as the “extrinsic” type^3,4^. Human atopic dermatitis has been regarded as type 2 dominated characterized by interleukin (IL)-4, IL-5, and IL-10 producing T cell, and high IgE antibody titers. However, recent studies have found interferon (IFN)-γ producing CD8^+^ cells supporting theories of a mixed type 1 and type 2 immune response^5^. In a frequently used mouse model, induction of delayed type 1 hypersensitivity is obtained by topical application of haptens such as oxazolone, which cause secretion of proinflammatory cytokines (IL-1β, TNF-α) by keratinocytes^6^. Frequent oxazolone challenges (nine-ten times) can provoke a shift from the typical type 1 immunity reaction to a chronic type 2 inflammatory response in mice resembling the mixed response seen in human atopic dermatitis^7^.

Atopic dermatitis is a polygenic disease, but the increasing prevalence suggests environmental factors contributing to disease development^8^. Previously, we have reported a strong correlation between gut microbiota composition and inflammatory skin response in an oxazolone-induced dermatitis model in BALB/c mice^9^, and we have found that a high- and low-responding phenotype can be transferred with the gut microbiota to germ-free mice^10^. This is in line with another study which demonstrated that an antibiotic treatment favored induction of regulatory T cells and suppressed ear swelling in a murine contact sensitivity model. The phenotype was also transferable with the gut microbiota^11^. Several studies in children show differences in the gut microbiota composition before and after clinical signs of atopic disease. A higher incidence of allergy was e.g. evident in children with low proportions of *Bifidobacterium* spp. and children colonized with *Clostridium* spp.^12–15^. Additionally, bifidogenic manipulation of the gut microbiota composition in the first year of life by human milk oligosaccharides has shown preventive effects on atopic dermatitis and allergies^16^. The bifidogenic effect of feeding fructo-oligosaccharides (FOS) has also been found to reduce ear swelling and IFN-γ production in a 2,4-dinitrofluorobenzene-induced contact hypersensitivity model in mice, while increasing anti-inflammatory IL-10 production in the draining lymph node ^17^. FOS belongs to a group of carbohydrates called prebiotics, defined as dietary components promoting specific changes in the composition and activity of the gut microbiota beneficial for host health^18^. Another prebiotic xylooligosaccharide (XOS) approved for human consumption is composed of monomers of xylose units, and has been reported to propagate lactobacilli in feces in the same way as FOS^19,20^. We have previously seen that dietary XOS also significantly increased *Bifidobacterium* spp*.* throughout the intestines and down-regulated IFN-γ and IL-1β blood levels in mice^21^.

In the present study, we wanted to further address the anti-inflammatory potential of dietary XOS and a possible alleviating effect on disease in an oxazolone-induced dermatitis model. High-throughput sequencing of the gut microbiota was applied to discover bacteria with anti-inflammatory potential.

## Methods

### Animals, housing, and experimental design

The experiment complied with the Danish Act on Animal Experimentation, which implements the Directive 2010/63/EU on the Protection of Animals used for Scientific Purposes. It was approved by the Danish Animal Experiments Inspectorate at Ministry of Environment and Food (License number: 2012-15-2934-00399), and all the studies were conducted according to the NIH guide for the Care and Use of Laboratory Animals.

32 four weeks old female BALB/cJBomTac mice (Taconic, L. Skensved, Denmark) arrived to our barrier protected facility (room temperature 22 ± 2 °C, relative humidity 55% ± 10%, air changes 15–20 times per hour, lighting interval 06.00 a.m.—06.00 p.m.) as ”Murine Pathogen Free”, and housed according to FELASA recommendations^22^. The animals were randomly divided into two groups of four cages each fed either Altromin 1430 control diet or Altromin 1430 diet modified with 47.5 g XOS/kg ad libitum (Altromin, Brogaarden, Lynge, Denmark) throughout the experiment. 10% of the total content of saccharides consisted of XOS (XOS DP 2–6, Shangdong Longlive, Qingdao, China). According to the product label, the Nitrogen-Free Extract (NFE) of the two diets were 427.5 g NFE/kg and 475 g NFE/kg in the XOS and control diet, respectively. All animals had free access to untreated tap water in bottles. Food and water consumption per cage were measured weekly. Bedding consisted of aspen chips, and all cages were supplied with a cardboard house, a chewing aspen block, a mini fun tunnel, and a Nestlets felt pillow (all delivered by Brogaarden).

**Figure 1:**
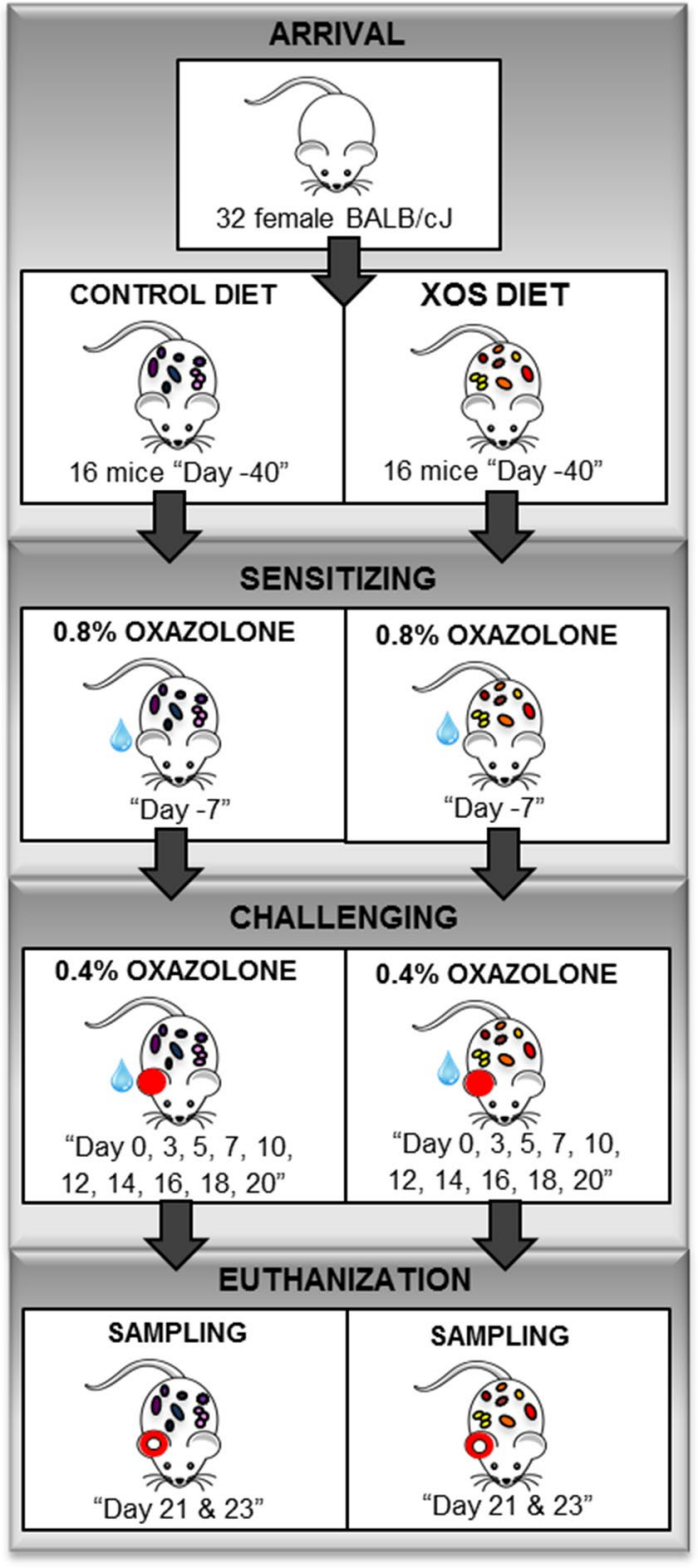
Experimental design. 32 female BALB/cJ mice at weaning were randomly divided into two groups fed either a standard control diet or a xylooligosaccharide (XOS) supplemented diet. After 33 days, the mice were sensitized with 0.8% oxazolone before they were challenged ten times with 0.4% oxazolone solution on the right ear. The mice were euthanized after the last challenge and sampled for further analyses.

Induction of atopic dermatitis was initiated by sensitizing all animals on Day -7 with oxazolone solution containing 0.8% oxazolone (w/v %) (4-ethoxymethylene-2-phenyl-2-oxazolin-5-one, E0753, Sigma-Aldrich, St Louis, MO) and 4:1 acetone (Emsure, MerckChemicals, Darmstadt, Germany) and oil (Organic extra virgin olive oil, Svansø, Scandic Food A/S, Vejle, DK). A 0.4% oxazolone solution was used for challenges on Day: 0, 3, 5, 7, 10, 12, 14, 16, 18, and 20 (Fig. 1). Applications were performed by distributing 20µL oxazolone solution on the medial and lateral sides of the right pinna of each animal for both sensitization and challenges. Every second day of challenge the control group was treated before the XOS group and vice versa.

### Ear thickness and clinical skin inflammation score

Ear thickness was measured in vivo the day of sensitizing (Day-7), and the final ear thickness was measured on sedated mice twice by the same person immediately before euthanasia (Mitutoyo Low Force Caliper Series 573, Aurora, IL). Dermatitis was evaluated post mortem independently by two blinded persons applying a clinical skin inflammation score. The inflammation was evaluated on (i) redness, (ii) thickening, (iii) excoriation/erosion, and (iv) incrustation. Scoring grades were assigned for each of the four parameters as follows: 0 = no sign; 1 = mild; 2 = moderate; 3 = severe, modified after Fujiwara et al.^23^, resulting in a total score up to 12. The average total score was calculated for each animal.

### Histology

Paraffin blocks of the formalin fixed 3 mm ear tissue biopsies were cut at 2 µm cross sections and stained with hematoxylin and eosin (HE) or toluidine blue for mast cells. All samples were blinded and scored at 4x, 10x, and 40 × magnification several times by the same person: (i) epidermal spongiosis (intercellular edema), (ii) dermal hyperplasia, (iii) mast cell infiltration, and (iv) fibroplasia were separately given a score from 0 to 3 (0 = none; 1 = minimal; 2 = moderate; 3 = severe) resulting in a total score up to 12. Mast cells were counted in two High Power Fields (40 × magnification) per sample, one on each side of the cartilage, and a score from 0 to 3 was given based on the mean of the two counts (0 ≤ 1 mast cell; 1 = 1–5 mast cells; 2 = 6–10 mast cells; 3 ≥ 10 mast cells).

### Serum IgE

Serum was collected from anaesthetized animals before decapitation, and IgE levels were measured using Mouse IgE ELISA Kit (Bethyl Laboratories, Montgomery, TX) according to the standard protocol using a 1:20 dilution. Absorbance was measured and analyzed (PowerWave x Microplate Spectrophotometer and KC4 v3.4, Rev 21, Bio-Tek Instruments INC, Winooski, VT).

### Ear tissue cytokines

An 8 mm punch biopsy (KRUUSE, Langeskov, Denmark) was taken from the right ear of all animals. From the center of the 8 mm biopsy, a 3 mm punch biopsy (KRUUSE) was collected and stored in 4% formaldehyde (Hounisen, Skanderborg, Denmark). The remaining ring-shaped biopsy was snap-frozen in liquid nitrogen. The day before analysis, the samples were homogenized by blending (POLYTRON PT1200E Manual Disperser, Kinematica AG, Luzern, CH) in 300µL lysis buffer (Meso Scale Discovery, Rockville, MD) and stored at 4 °C. Levels of mouse IFN-γ, IL-1β, IL-2, IL-4, IL-5, CXCL-1, IL-10, IL-12p70, and TNF-α were measured using MSD MULTI-SPOT Assay System, Proinflammatory Panel 1 (mouse) kit (V-PLEX, MSD) on a MESO QuickPlex SQ120 and analyzed by the standard software Discovery Workbench v4 (MSD).

### Ear tissue gene expression

Homogenization of ear biopsies, RNA isolation using MagMAX-96 RNA Isolation Kit (Ambion, Austin TX), and cDNA synthesis using High-Capacity cDNA Reverse Transcriptase Kit (Applied Biosystems, Foster City, CA) were performed as described previously^21^. qPCR analyses using *Foxp3, Tgfβ, Il4* and *Tnf* TaqMan gene expression assays and *Actinβ* as reference gene (Applied Biosystem) were done to assess local regulatory T cell function and inflammation in the ear. Data were analyzed as described previously^21^. Statistical comparison was done on ΔC_T_ values.

### Flow cytometry

A single-cell suspension was made by squeezing the right superficial parotid lymph node and suspending the cells in PBS from all animals immediately after euthanasia. The cell suspension was passed through a 100 µm nylon mesh. Samples were stained with either (i) CD3, CD8α, CD19b, and NKT or (ii) CD4, CD103, and intracellular FoxP3 according to manufacturer’s protocol (eBiosciences, San Diego, CA). Samples were analyzed using a BD Accuri C6 cytometer and BD FLOW CYTOMETRYDIVA software (Accuri Cytometers Inc., Ann Arbor, MI). The gating strategy is outlined in Supplementary Fig. S1.

### Gut microbiota

The fecal microbiota composition was determined for ten mice per group before (Day-7) and after (day of euthanasia) oxazolone-induced disease using tag-encoded 16S rRNA gene amplicon MiSeq-based high throughput sequencing (Illumina Inc., San Diego, CA). Feces were collected from each mouse in a sterile eppendorf tube and stored at -80 °C. DNA extraction, storage, and sequencing library preparation steps were conducted as previously described^24^ with following modifications: For the first PCR step, primers targeting the V3 hypervariable region of 16S rRNA gene, containing additional adapters compatible with Nextera Index Kit (Illumina Inc.), were used. The primer sequences were as follows: NXt_388_F:5′TCGTCGGCAGCGTCAGATGTGTATAGAGACAGACWCCTACGGGWGGCAGCAG-3′ and NXt_518_R:5′-GTCTCGTGGGCT CGGAGATGTGTATAAGAGACAGATTACCGCGGCTGCTGG-3′. Sequencing was performed using the V2 kit (Illumina Inc.). The raw dataset containing pair-ended reads with corresponding quality scores was truncated to 150 bp, merged, and trimmed using fastq_mergepairs and fastq_filter scripts implemented in the UPARSE pipeline^25^. The minimum overlap length was set to 150 bp. The minimum length of merged reads was 150 bp. The maximum expected error was E = 2.0, and the first truncating position with quality score was N ≤ 4. Purging the dataset from chimeric reads and constructing de novo Operational Taxonomic Units (OTU) were conducted using the UPARSE pipeline^25^. The Green genes (13.8) 16S rRNA gene collection was used as a reference database^26^. Quantitative Insight Into Microbial Ecology (QIIME) open source software package (1.7.0 and 1.8.0) was used for subsequent analysis steps^27^.

Principal coordinate analysis (PCoA) plots were generated with the Jackknifed Beta Diversity workflow based on 10 distance metrics calculated using 10 subsampled OTU tables. The number of sequences taken for each jackknifed subset was set to 85% of the sequence number within the most indigent sample (50,000 reads/sample). Analysis of similarities (ANOSIM) was used to evaluate group differences using weighted and unweighted uniFrac distance metrics^28^ that were generated based on rarefied (50,000 reads/sample) OTU tables. The differences in taxa abundance between the groups were estimated with a statistic framework: analysis of composition of microbes (ANCOM) based on non-normalised OTU-table summarized to the species level with abundance threshold 0.1%.

### Statistics

Gaussian distribution tests (D’Agostino & Pearson omnibus, or Shapiro–Wilk normality test if n < 5) were applied to all quantitative data and analyzed using unpaired t-test with Welch’s correction or Mann–Whitney U-test if n < 5. ANOVA was used to exclude any differences between cages within each group. For clinical and histopathology score a point of reference equal to the 75% percentile of frequency distribution in the control group divided the animals in below ( >) or on/above ( ≤) point of reference. These dichotomous data were analyzed with Chi-square (χ^2^-test). All statistical analysis and graph designs were made in GraphPad Prism version 6 (La Jolla, CA).

## Results

### Oxazolone treatment induced severe dermatitis on the ears of mice

**Figure 2:**
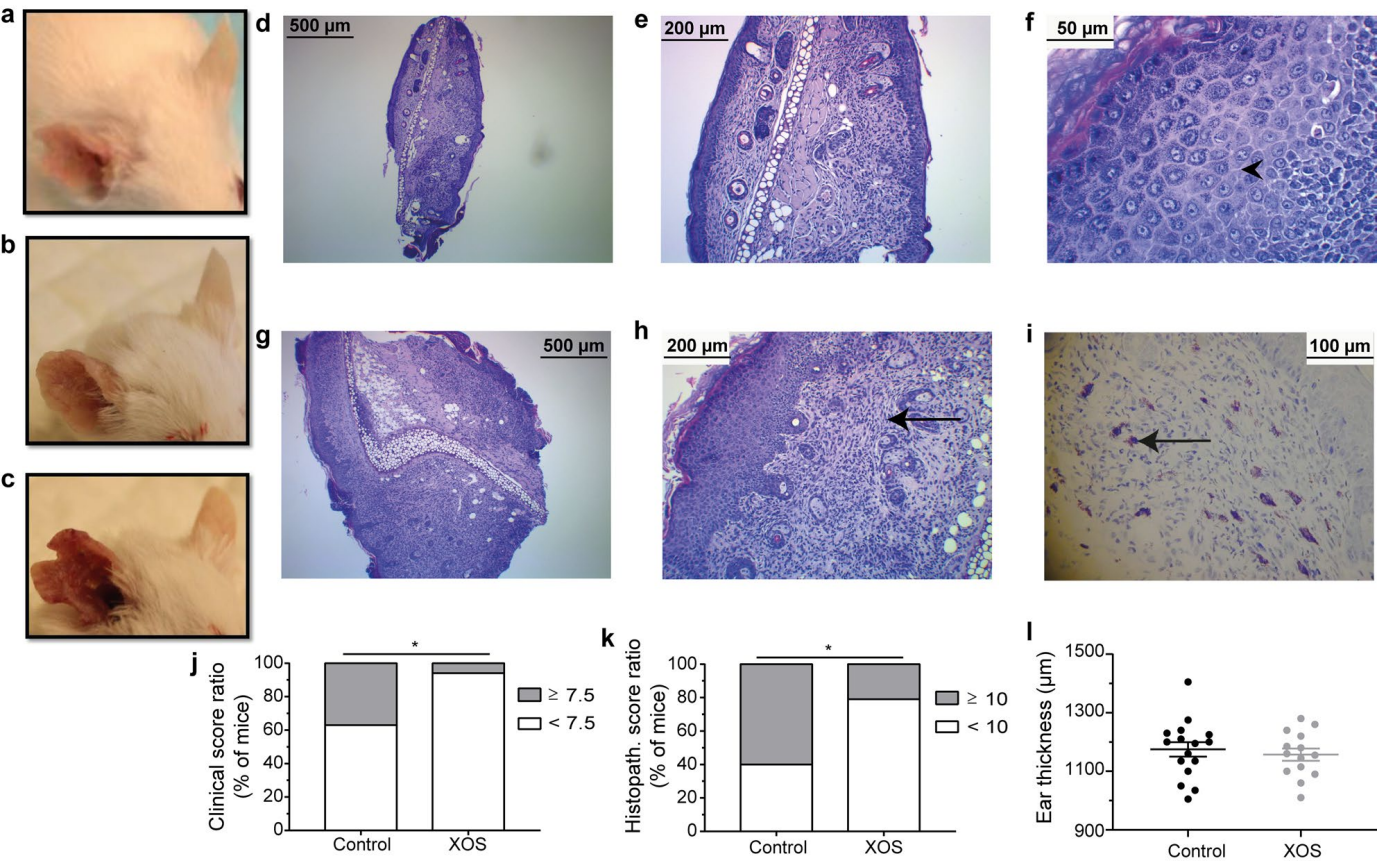
Dietary XOS reduced the ratio of high-responding mice. Photos of mouse ears with (**a**) mild, (**b**) moderate, and (**c**) severe oxazolone-induced dermatitis are shown. (**d–e**) Representative HE-stained cross sections of an inflamed ear with a low total histopathological score of 7 shown at 4 × and 10 × magnification. (**f–h**) 4 ×, 10 ×, and 40 × magnification of a representative cross section from a high responder with a total histopathological score of 10. Fibroplasia (arrows) and spongiosis (arrowheads) are seen. (**i**) Representative toluidine blue stained cross section from a high responder is shown at 10 × magnification. Mast cells (arrows) are seen. The degree of oxazolone-induced dermatitis was clinically scored by two blinded persons independently in mice fed either control (*n* = 16) or XOS (*n* = 16) diet when euthanized. (**j**) The percentage of mice expressing a high (average total score ≥ 7.5) and low (average total score < 7.5) phenotype is shown. **k**) Cross sections of the inflamed ears were scored blindly in mice fed control (*n* = 15) or XOS (*n* = 14) diet. The percentage of mice expressing a high (total score ≥ 10) or low (total score < 10) phenotype is shown. (**l**) Ear thickness was measured twice on anaesthetized mice fed control (*n* = 16) or XOS diet (*n* = 16). The average ear thickness is shown. * indicates *p* < 0.05.

In this study, BALB/cJBomTac mice were fed either XOS supplemented or control diet and atopic dermatitis was successfully induced in all mice with repeated 0.4% oxazolone solution treatments. The oxazolone treatment increased ear thickness on the treated ears from 777 ± 14 µm and 778 ± 14 µm on Day -7 to 1175 ± 100 µm and 1157 ± 78 µm at euthanasia in the control and the XOS group, respectively (mean ± s.d.; p < 0.0001). Clinical symptoms of acute inflammation characterized by disseminated redness, dermal thickening, and nociceptive behavior were observed in all mice from Day 5 and throughout the experiment. Acute dermatitis with loss of skin barrier function characterized by focal or multifocal distributed thin, translucent, and moist skin lesions (erosions) was observed until approximately Day 16. Then skin repair became predominant with clear visual signs of wound healing still combined with thickening of the ear, erosions, and redness until euthanasia (Fig. 2a–c). Microscopic examination of ear tissue biopsies revealed epidermal and dermal hyperplasia in all samples (Fig. 2d + g). Epidermal inflammation was characterized by hyperplastic stratum spinosum, spongiosis and exocytosis, hypergranulosis, and parakeratosis (Fig. 2f + h). Moderate to severe dermal histiocytic infiltration and varying grades of fibroplasia were found (Fig. 2e + h), as well as fully granulated mast cells (Fig. 2i). In addition, pustules, crusts, and erosions were frequent but random findings. Hyperkeratosis was present in all samples, as well as other findings indicating high tissue turnover, but stratum corneum was lost in most slides.

### A XOS supplemented diet reduced the number of high responders

**Figure 3:**
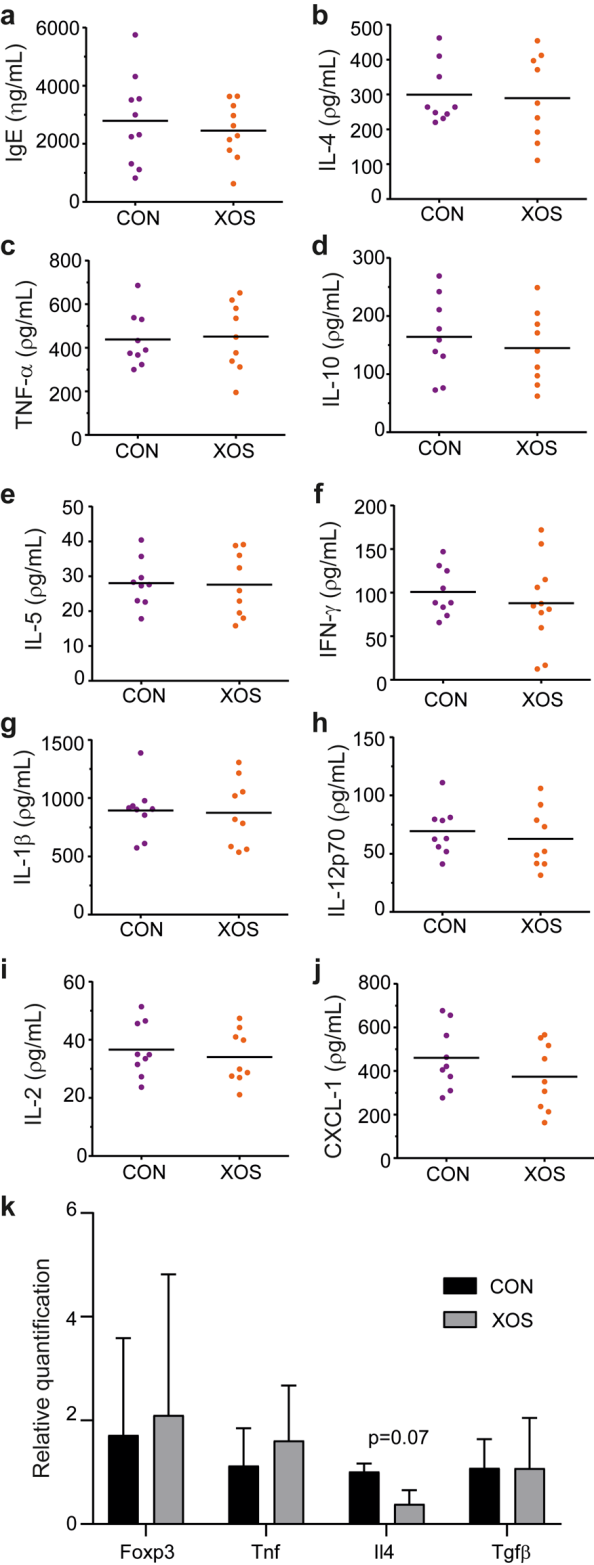
Serum IgE and ear tissue cytokine levels were not sensitive to dietary regulation. (**a**) Serum IgE was measured with ELISA in mice with oxazolone-induced dermatitis fed either XOS or control diet (CON) at euthanasia (*n* = 10/group). (**b–j)** Cytokines were measured with a mesoscale multiplex kit in punch biopsies sampled from the inflamed ear (*n* = 9/group). The mean is shown for each cytokine as indicated. (**k**) Gene expression of anti-inflammatory regulatory T cell markers (*Foxp3* and *Tgfβ*) and proinflammatory cytokines (*Il4* and *Tnf*) were analyzed by qPCR on RNA extracted from ear tissue punch biopsies (*n* = 4/group). Mean and s.d. are shown.

Clinical evaluation of excoriation, erosion, and incrustation showed focal or multifocal dissemination with high inter-animal variation in both groups (Table 1). Only one mouse in the XOS group received a high clinical skin inflammation score above the upper 75% percentile, compared to six mice in the control group (scores 7.5–9.5; p < 0.05; Fig. 2j). Similarly, histological evaluation of spongiosis, hyperplasia, mast cell infiltration, and fibroplasia displayed a significant variation (Table 1), and significantly fewer mice in the XOS group (3 out of 14) were given a high histopathological score above the 75% percentile compared to the control group (9 out of 15; p < 0.05; Fig. 2k). There was no difference in ear thickness at euthanasia between the two dietary groups (Fig. 2l). Serum IgE concentration (Fig. 3a), ear tissue cytokine levels (Fig. 3b–j) and gene expression of anti- and proinflammatory markers (Fig. 3k), as well as immune cell composition in the draining lymph node (Supplementary Fig. S2) were in average similar in the two dietary groups, but a large variation was also evident in several of these parameters further indicating high and low responders to the oxazolone treatment. Thus, an analysis was performed to evaluate correlation between immunological parameters as well as clinical and histological scores in each dietary group (Supplementary Table S1). The clinical ear inflammation score tended to correlate with ear thickness and total histological score in both the control group and in the XOS group. The clinical score also correlated strongly with serum IgE and all the local cytokine levels, supporting the notion of high and low responders to oxazolone treatment in the control group in contrast to the XOS fed group which consisted primarily of low responders. Neither NFE intake nor body weight was different between groups (Supplementary Fig. S3), demonstrating that the effect of feeding prebiotics was not related to altered metabolisable energy intake, but was more likely microbiota-mediated.

**Table 1:** Total clinical score and histological score of XOS and control fed mice with oxazolone-induced dermatitis.

Total clinical score	N (%) – Control^1^	N (%) – XOS^1^
2	1 (6.25%)	0 (0%)
3	1 (6.25%)	1 (6.25%)
3.5	1 (6.25%)	0 (0%)
4	2 (12.5%)	2 (12.5%)
4.5	0 (0%)	1 (6.25%)
5	1 (6.25%)	2 (12.5%)
5.5	3 (18.75%)	1 (6.25%)
6	0 (0%)	1 (6.25%)
6.5	1 (6.25%)	3 (18.75%)
7	0 (0%)	4 (25)
**7.5**	**3 (18.75%)**	**1 (6.25%)**
**8**	**1 (6.25%)**	**0 (0%)**
**8.5**	**1 (6.25%)**	**0 (0%)**
**9**	**0 (0%)**	**0 (0%)**
**9.5**	**1 (6.25%)**	**0 (0%)**

Total histo score	N (%) – Control^2^	N (%) – XOS^2^
6	0 (0%)	1 (7.14%)
7	2 (13.3%)	3 (21.4%)
8	2 (13.3%)	3 (21.4%)
9	2 (13.3%)	4 (28.6%)
**10**	**6 (40%)**	**2 (14.3%)**
**11**	**3 (20%)**	**1 (7.14%)**

^1^The number of mice for each clinical score is given as well as the percentage out of the total number of mice. N = 16 per dietary group. ^2^The number of mice for each histological score is given as well as the percentage out of the total number of mice. N = 15 in the control and n = 14 in the XOS fed group. The high responder scores are all marked in bold.

### *Prevotella* spp. were associated with a low-responding phenotype and sensitive to dietary change

**Figure 4:**
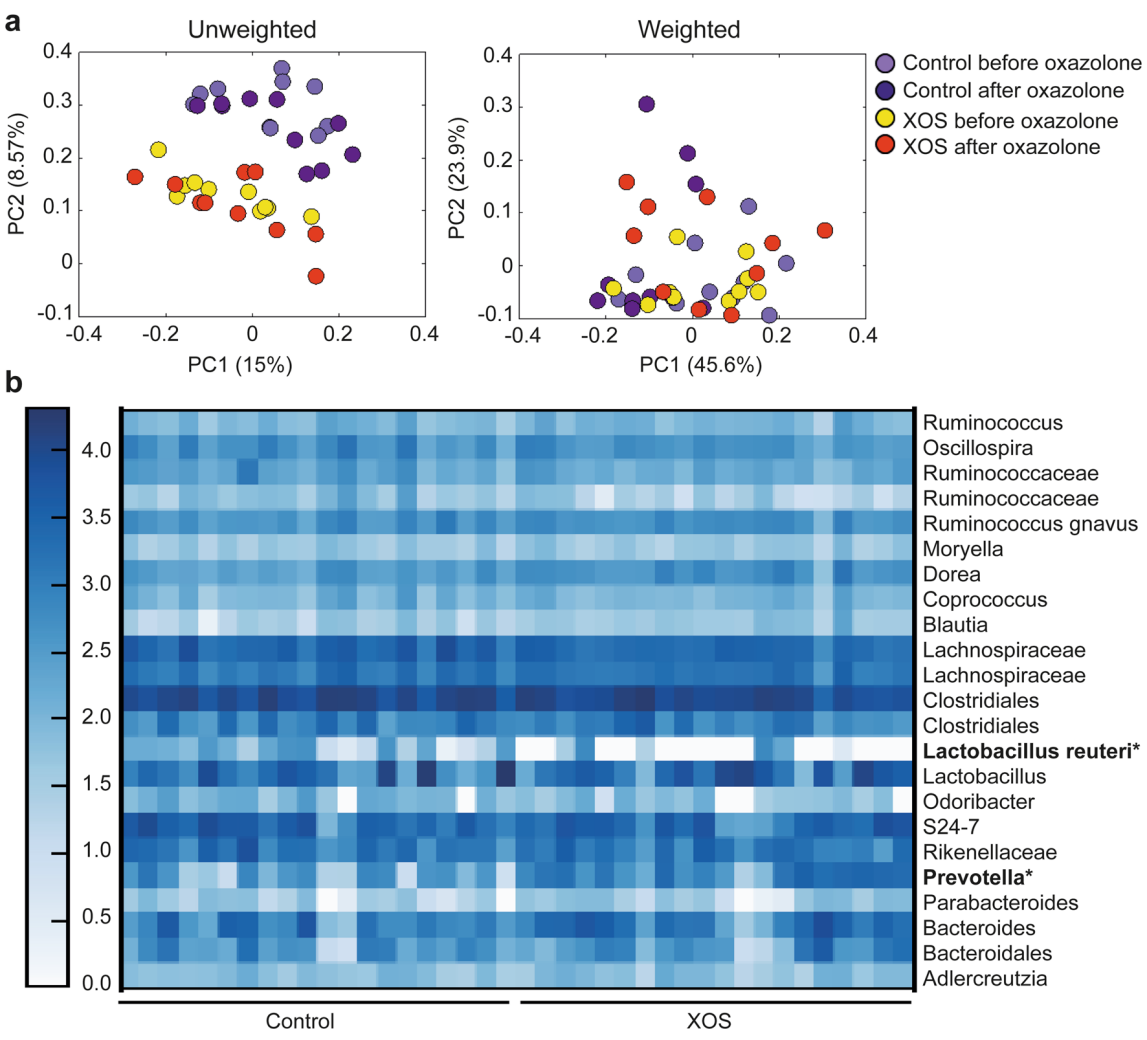
XOS diet promoted *Prevotella* spp. and reduced *Lactobacillus reuteri.* (**a**) Principal coordinates analysis plot of 16S rRNA gene tag-encoded amplicon sequencing based on the unweighted and weighted UniFrac distance matrix are shown as indicated. The plots illustrate feces samples from the same mice (*n* = 10/group) before and after oxazolone challenge fed either control (light and dark purple, respectively) or XOS supplemented diet (yellow and red respectively). (**b**) Heat map showing the relative abundance of the most abundant taxa examined using 16S rRNA gene (V3 region) amplicon sequencing in feces samples collected before and after oxazolone treatment. Bacterial taxa reported as significantly different (ANCOM *p* < 0.05) between the dietary groups are bold and marked with *. The analysis was performed using non-normalized, summarized to species level OTU-table with abundance threshold 0.1%.

**Figure 5:**
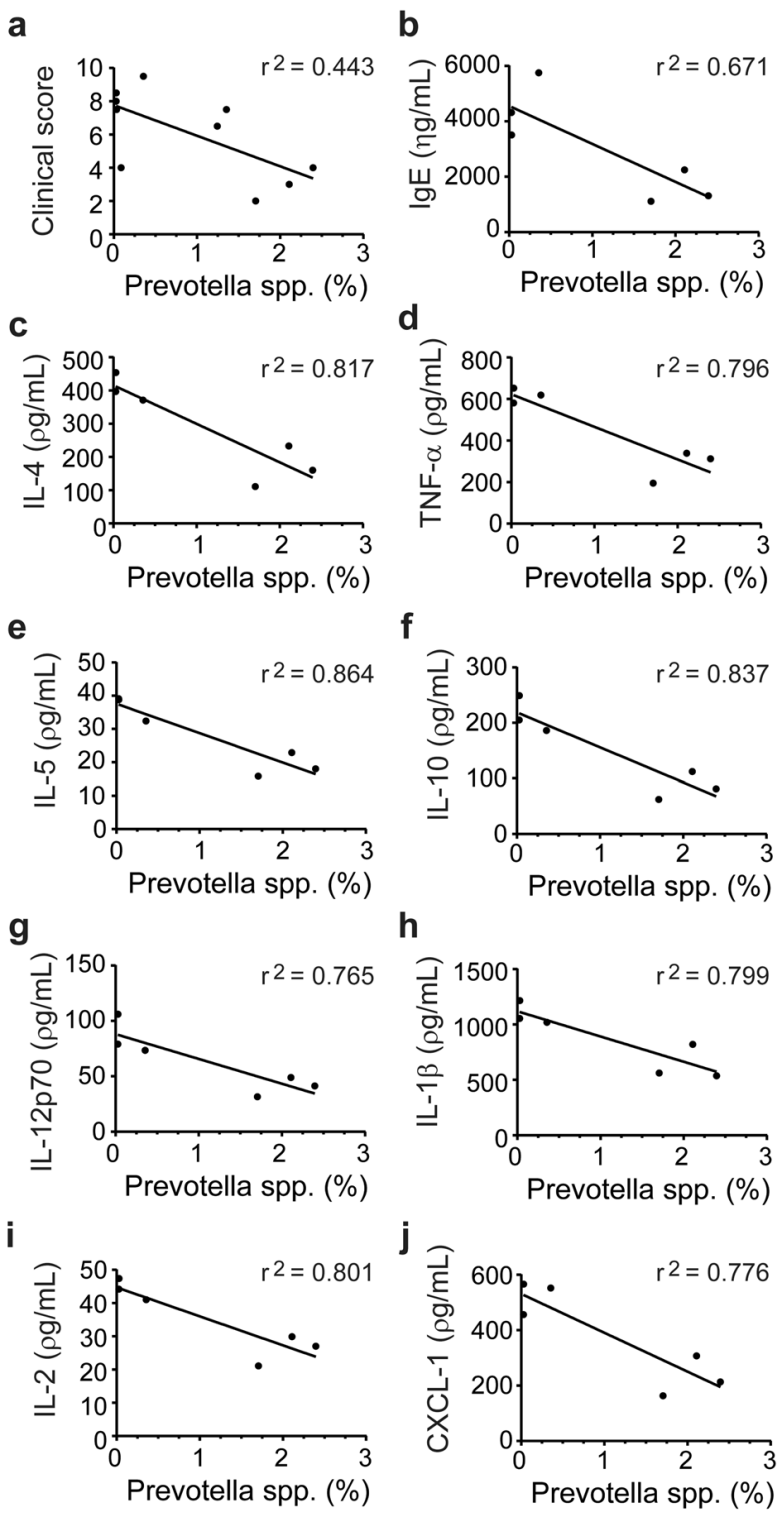
*Prevotella* spp. associated with markers of a high-responding phenotype. Significant correlations were found between the abundance of OTUs annotated as *Prevotella* and the clinical ear inflammation score (**a**, *n* = 10), serum IgE levels (**b**), IL-4 (**c**), TNF-α (**d**), IL-5 (**e**), IL-10 (**f**), IL-12p70 (**g**), IL-1β (**h**), IL-2 (**i**), and CXCL-1 (**j**) concentrations in biopsies from the inflamed ear of mice on control diet and oxazolone-induced dermatitis (*n* = 6). Feces samples were collected on the day of euthanasia and analyzed by 16S rRNA gene (V3 region) amplicon sequencing. Phenotypic markers used to characterize the high and low-responding phenotypes were all collected at euthanasia. Serum IgE was analyzed by ELISA, and the cytokines were analyzed by mesoscale multiplex kit. *P* < 0.05 for all correlations and r^2^ values for the linear regression are shown.

Sequences from the microbiota analysis purged from chimeric reads yielded in average 170,000 sequences per sample. The XOS fed mice clustered separately from the control fed mice on the unweighted Principal coordinate analysis (PCoA) both before and after oxazolone treatment (ANOSIM p < 0.001 and R = 0.36), while no separate clustering was evident in the weighted PCoA (Fig. 4a). Neither the oxazolone treatment nor cage contributed to clustering of the gut microbiota. Consequently, none of these were considered confounding factors in the microbial analyses. Differences between mice fed XOS supplemented and control diet were mainly driven by higher abundance of *Prevotella* spp. and a decreased abundance of *Lactobacillus reuteri* in the XOS fed mice compared to the control fed mice (Fig. 4b). None of the mice in either group harbored *Bifidobacterium* spp., a well-described target of oligosaccharides, making these mice useful to assess non-bifidogenic effects of the prebiotic. Of interest, the abundance of *Prevotella* spp. was not only modified by the diet but also correlated negatively with clinical ear inflammation score, serum IgE level, and cytokine levels in the control group (Fig. 5). In addition, the clinical ear inflammation score and serum IgE level correlated negatively with the much less abundant Enterococcaceae family and *Odoribacter* genus respectively in the control group (Supplementary Fig. S4), whereas no significant correlations were evident in the XOS group.

## Discussion

Modulation of the gut microbiota by dietary oligosaccharides alleviates sensitivity to oxazolone-induced dermatitis in mice. It has previously been shown that a FOS supplemented diet can reduce ear swelling in 2,4-dinitrofluorobenzene exposed adult NC/Nga mice^29^. The same group also investigated the bifidogenic effect of FOS by administering oral *B. pseudolongum* daily^17^, but the bifidobacteria only partially reduced ear swelling in control fed mice compared to the alleviating effect of FOS with or without bifidobacteria, suggesting that additional microbial pathways contribute to alleviation of disease by oligosaccharide supplemented diet. The alleviating effect was confirmed in our study, albeit differences in choice of mouse strain, prebiotic, hapten, and induction protocol. A XOS supplemented diet reduced the number of high responders to oxazolone treatment in BALB/c mice, but a large inter-individual variation in the response suggested that the alleviating effects were dependent on specific changes in the gut microbiota and, thus, evident only in mice with a given microbial profile. The low-responding phenotype was indeed associated with a high abundance of *Prevotella* spp. It is possible that *Prevotella* spp. just propagated in response to more severe disease and was not responsible for the effects of XOS, but XOS propagated *Prevotella* spp. before induction of disease which, thus, suggests the opposite scenario. *Prevotella* spp. is also a known fiber-utilizing bacteria in the human microbiome, propagated by high levels of complex carbohydrates, which makes it a promising target for microbiota-directed therapeutics such as prebiotics in atopic patients. However, it is important to stress that different human enterotypes respond differently to oligosaccharides, causing distinct profiles of short chain fatty acids that may impact host health differentially^30^.

Also in mice, different microbial profiles yield varying results of feeding XOS. In our previous study, dietary XOS had no effect on *Prevotella,* but increased *Bifidobacterium* spp. throughout the intestine, and down-regulated proinflammatory cytokines systemically^21^. Mice in the present study, did not harbor bifidobacteria, but other bacteria, able to utilize the prebiotic compound, was propagated. This enabled us to study other non-bifidogenic effects of prebiotics. Surprisingly, none of the immunological analyses showed any significant impact of XOS on the local cytokine environment, serum IgE, or immune cell populations in the draining lymph nodes. It is likely that (i) the strong immunogenic effects previously evident in our studies, when feeding XOS supplemented diet to mice, were attributed to its bifidogenic effects. Sasajima et al. have e.g. demonstrated that oral supplementation of *Bifidobacterium* spp. decrease IFN-γ production in lymph nodes to the same extent as a FOS supplemented diet^17^. It is also possible that (ii) XOS might show stronger anti-inflammatory effects if fed earlier in life e.g. by maternal feeding. In another study, offspring of NC/Nga mice consuming dietary FOS during gestation resulted in greater suppression of the spontaneously developing manifestation of skin inflammation, including lower expression of local TNF-α mRNA and serum antibody concentration compared to pups fed FOS after weaning^23^. (iii) In the present study, a standard model of atopic dermatitis was used with a dosage and frequency of oxazolone which may have overruled more subtle anti-inflammatory effects of the diet or gut microbiota, and consequently diminished group differences. It may therefore be beneficial in future studies to adjust the induction protocol to obtain less severe inflammation. Lastly (iv), propagating *Prevotella* spp. by feeding XOS can only be expected to influence the development of atopic dermatitis in a group of mice with many high responders containing low abundance of *Prevotella* spp. in their gut. The difference between the dietary groups will as such become less visible in a group of mice with high variation in the abundance of *Prevotella* spp., which could explain the lack of difference in many of the immunological parameters that otherwise correlated strongly to the clinical phenotype. Testing the effect of prebiotics in mice without *Prevotella* spp. (and/or bifidobacteria) would be beneficial to predict the effect of prebiotics on individuals with atopic dermatitis hosting different enterotypes^31^. In general, more research, that aims to identify specific microbes involved in regulating atopic disorders, is necessary to enable stratified treatments dependent on the individual gut microbial profiles.

In general, *Prevotella* spp. have been shown to exhibit proinflammatory properties, e.g. *P. copri* has Th17 promoting capabilities^32^ and has been correlated with the development of rheumatoid arthritis in humans^33^. It can also enhance Th1 immune responses in dextran sulfate sodium-induced colitis in mice^34^. Caspase-3 knockout mice, which normally exhibit a lower inflammatory response to DSS induction of colitis compared to wild-type mice, can have this protective effect diminished if their abundance of *Prevotella* spp. is increased by cohousing with wild-type mice^35^. In the gut of leptin-deficient obese mice a high abundance of Prevotellaceae correlates with an impaired glucose tolerance^36^, and increased abundance of *P. copri* was also linked to insulin-resistance in humans^37^. On the other hand, *Prevotella* spp. dominate the healthy lung of humans compared to patients with asthma and chronic obstructive pulmonary disease^38^, and reduce inflammatory cytokines in cells from mouse lungs compared to bacteria isolated from asthma patients^39^. Thus, *Prevotella* spp. could play a protective role in Th2 mediated disease as supported by the present study, but more studies are needed for clarification. In addition, *L. reuteri* was downregulated by the XOS diet, in the present study, but was neither associated with the high- nor the low-responding phenotype. The presence of *L. reuteri* in the first week of life has previously been associated with low Th2 response in children^40^, but the alleviating effect of dietary XOS on atopic dermatitis was, in the present study, solely ascribed to *Prevotella* spp.

In conclusion, dietary oligosaccharides have the potential to diminish atopic dermatitis also in hosts without *Bifidobacterium* spp. In particular, intestinal *Prevotella* spp. were promoted by a prebiotic supplemented diet, and correlated strongly with a low inflammatory response to oxazolone-induced dermatitis i.e. less skin inflammation, lower serum IgE, and a reduced production of local inflammatory cytokines. It would be interesting to investigate the alleviating effects of *Prevotella* in other models of allergic disease, and its abundance should be considered when studying such diseases in animal models due to its major impact on variation. The role and mechanisms of intestinal *Prevotella* in relation to allergy should be studied further, in hope of providing better treatment strategies for atopic patients.

## Supplementary information

Supplementary Information 1.

## Data Availability

The datasets generated and/or analyzed during the current study are available from the corresponding author on reasonable request.
