# Supplementary material for: Dietary prebiotics promote intestinal Prevotella in association with a low-responding phenotype in a murine oxazolone-induced model of atopic dermatitis
Source: Sci Rep. 2020 Dec 3;10:21204. doi: 10.1038/s41598-020-78404-0 (PMC7713185; doi:10.1038/s41598-020-78404-0)
Supplement: Supplementary file 1 — Supplementary Information 1. [file 41598_2020_78404_MOESM1_ESM.pdf]

Dietary prebiotics promote intestinal *Prevotella* in association with a low-responding phenotype in a murine oxazolone-induced model of atopic dermatitis

Ann Laigaard, Lukasz Krych, Line F. Zachariassen, Lea Ellegaard-Jensen, Dennis S. Nielsen, Axel K. Hansen, Camilla H. F. Hansen

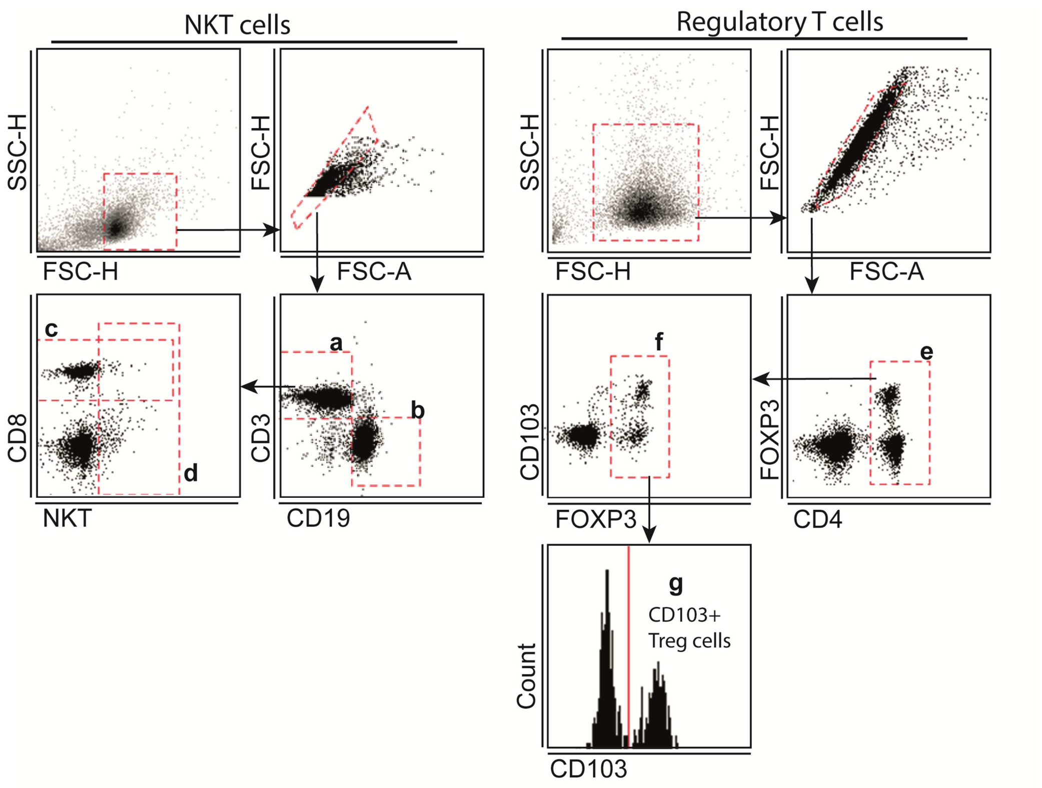

Figure S1. **Flow cytometric gating strategies**
Flow cytometric analysis of lymphocytes isolated from the right superficial parotid lymph node from control or XOS fed mice with oxazolone induced atopic dermatitis. Plots illustrate an example of the gating strategies for (**a**) T cells, (**b**) B cells, (**c**) cytotoxic T cells, and (**d**) NKT cells, as well as (**e**) T helper cells, (**f**) regulatory T cells, and (**g**) memory regulatory T cells. The arrows indicate which gate the following population originates from. The letters correspond to letters in figure S2.

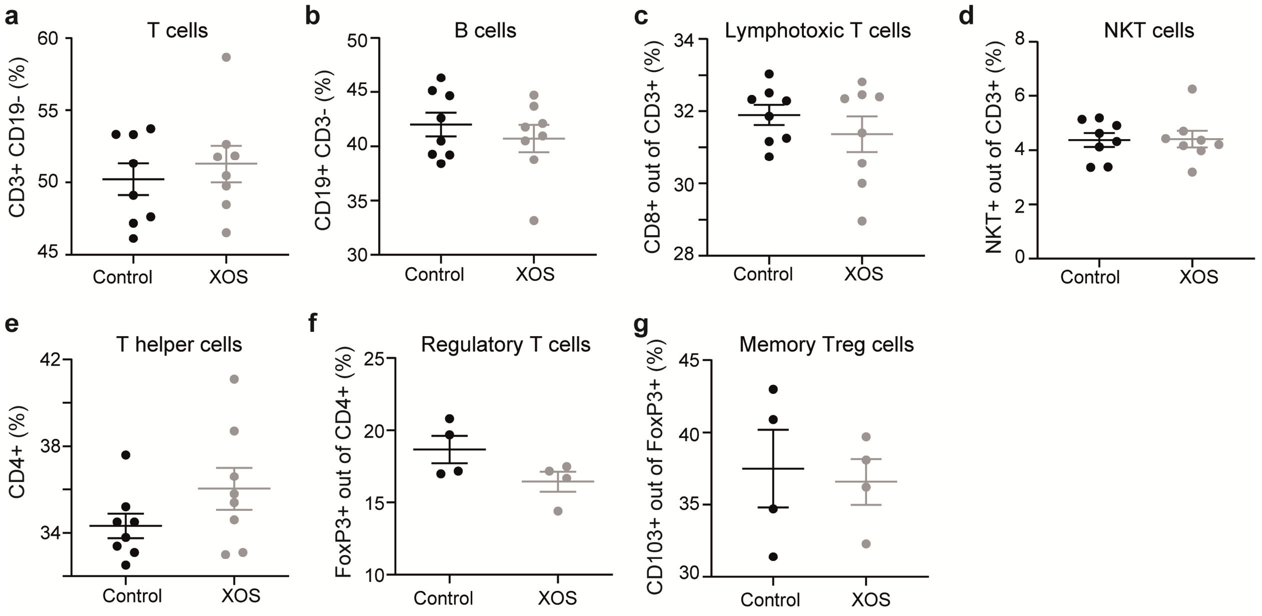

Figure S2. **Immune cell composition was not sensitive to dietary regulation**
Flow cytometric analysis of cells isolated from the right superficial parotid lymph node from control or XOS fed mice with oxazolone induced atopic dermatitis (n=8/group). The plots illustrate percentages of (**a**) CD3+ T cells, (**b**) CD19+ B cells, (**c**) CD8+ lymphotoxic T cells, and (**d**) NKT+ cells as well as (**e**) CD4+ T helper cells, (**f**) FoxP3+ regulatory T cells (n=4/group), and (**g**) CD103+ memory regulatory T cells (n=4/group). Gating as demonstrated in figure S1.

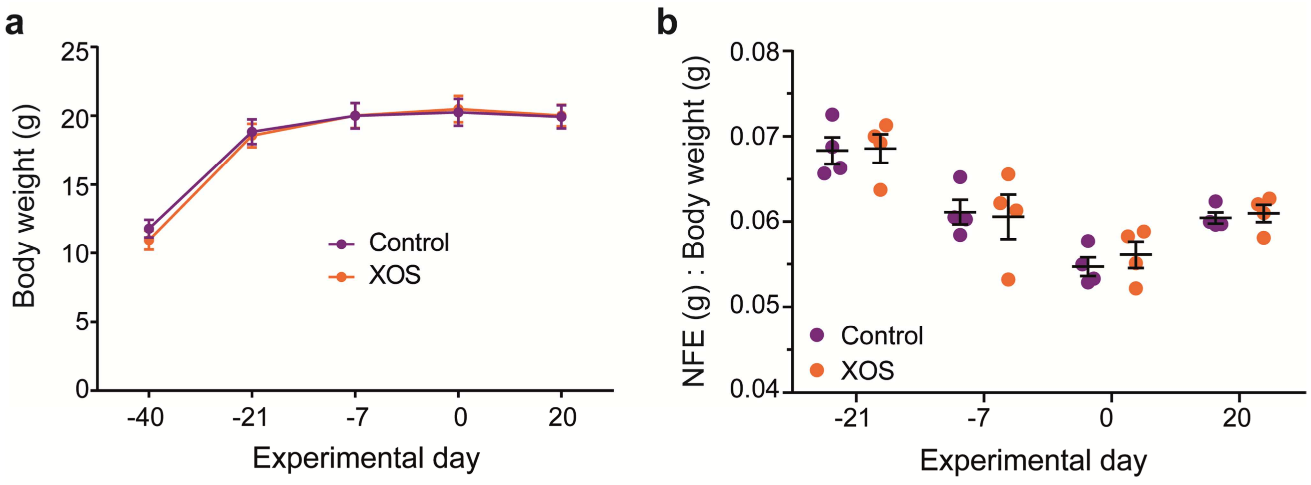

Figure S3. **Weight and food consumption were unaffected by diet** Body weight (**a**) and nitrogen-free extract consumption (**b**) in control and XOS fed mice with oxazolone-induced atopic dermatitis on the experimental days outlined in figure 1. Mean and SD are shown in **a** (*n*= 16 mice per group), mean and SEM are shown in **b** (*n*=4 cages per group). NFE calculations were based on cage level.

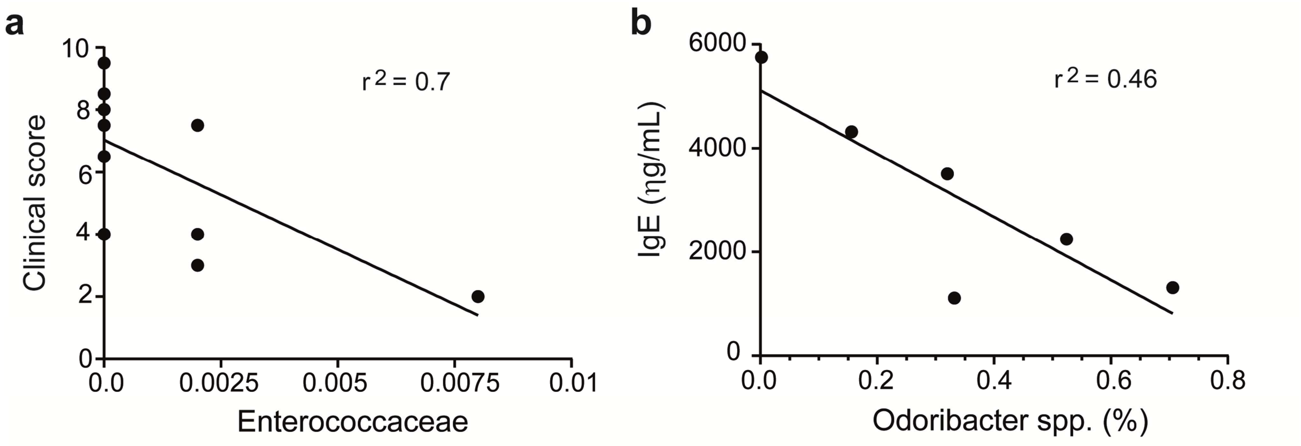

Figure S4. Enterococcaceae and *Odoribacter* spp. associated with markers of a high-responding phenotype

Significant correlations were found between the abundance of OTUs annotated as Enterococcaceae family and the clinical ear inflammation score (**a**, *n*=10) as well as between *Odoribacter* spp. and serum IgE levels (**b**, *n*=6). $p < 0.05$ for both correlations.

Table S1. Correlations of host parameters

P / r values[1]	Ear thickness	Clinical score	Histolog. score	Serum IgE	%CD3	%CD8/CD3	%CD19	%NK	%NKT	%CD4	%CD103/CD4	IFN-γ	IL-10	IL-12p70	IL-1ß	IL-2	IL-4	IL-5	CXCL-1	TNF-α
Ear thickness	-	0.5363	-0.0913	0.5762	0.8507	0.8732	-0.8414	-0.0920	-0.5347	-0.3294	-0.2359	0.4984	0.7342	0.7332	0.8154	0.7845	0.7887	0.7918	0.7837	0.8307
Clinical score	0.0890	-	0.4752	**0.8198**	0.2330	0.7064	-0.2066	-0.5549	0.2269	0.3351	0.3763	**0.8635**	**0.8642**	**0.7441**	0.6473	**0.8579**	**0.8175**	**0.7997**	**0.8441**	**0.9402**
Histolog. score	0.8020	0.0735	-	0.3689	-0.2357	-0.0214	0.2223	-0.5925	0.5057	0.5546	0.4250	0.4925	0.5576	0.4495	0.4070	0.4965	0.4375	0.4074	0.5348	0.5096
Serum IgE	0.2314	**0.0037**	0.2941	-	0.7566	0.7764	-0.7028	0.2553	0.0173	-0.8519	0.7740	**0.7545**	**0.7994**	**0.7148**	0.6494	**0.7827**	**0.8224**	**0.7035**	**0.8894**	**0.8537**
%CD3	0.0677	0.5788	0.5742	0.2434	-	0.4310	**-0.9926**	-0.2237	0.0195	-0.0374	-0.4628	-0.2744	0.9092	0.7046	0.6498	0.6262	0.8450	0.6553	0.8281	0.9377
%CD8/CD3	0.0532	0.0502	0.9599	0.2236	0.2863	-	-0.4496	-0.3690	-0.3705	-0.3725	0.4173	-0.3398	0.9435	0.8773	0.8570	0.8433	0.9450	0.8513	0.9292	**0.9804**
%CD19	0.0740	0.6236	0.5967	0.2972	**0.0000**	0.2637	-	0.2792	0.0219	0.1005	0.4129	0.1978	-0.8768	-0.6699	-0.6446	-0.6060	-0.8125	-0.6598	-0.7922	-0.9149
%NK	0.8830	0.1534	0.1217	0.7447	0.5943	0.3684	0.5031	-	-0.2158	-0.1029	-0.2575	-0.6363	0.0824	0.4151	0.2710	0.4094	0.2242	0.1754	0.2563	0.0183
%NKT	0.3532	0.5890	0.2010	0.9827	0.9635	0.3663	0.9589	0.6078	-	**0.8645**	-0.0058	-0.2679	-0.2483	-0.5298	-0.8221	-0.7278	-0.4038	-0.8644	-0.3747	-0.3558
%CD4	0.5884	0.4171	0.1537	0.1481	0.9300	0.3634	0.8128	0.8083	**0.0056**	-	-0.1386	0.4719	**-0.9765**	-0.9248	-0.8541	-0.8684	**-0.9821**	-0.8306	**-0.9729**	**-0.9955**
%CD103/CD4	0.7024	0.3582	0.2939	0.2260	0.2482	0.3037	0.3093	0.5381	0.9891	0.7435	-	**-0.9572**	0.5931	0.7147	0.4195	0.5863	0.6472	0.3055	0.6810	0.5044
IFN-γ	0.3928	**0.0027**	0.1780	**0.0188**	0.7256	0.6602	0.8022	0.3637	0.7321	0.5281	**0.0428**	-	0.6296	0.4498	0.4504	**0.6931**	0.5688	0.6486	0.6134	**0.7207**
IL-10	0.1578	**0.0027**	0.1188	**0.0097**	0.0908	0.0565	0.1232	0.9176	0.7517	**0.0235**	0.4069	0.0692	-	**0.9465**	**0.8244**	**0.9601**	**0.9703**	**0.9039**	**0.9435**	**0.9630**
IL-12p70	0.1587	**0.0215**	0.2248	**0.0304**	0.2954	0.1227	0.3301	0.5849	0.4702	0.0752	0.2853	0.2244	**0.0001**	-	**0.9222**	**0.9470**	**0.9713**	**0.9166**	**0.9407**	**0.9066**
IL-1ß	0.0925	0.0595	0.2770	0.0584	0.3502	0.1430	0.3554	0.7290	0.1779	0.1459	0.5805	0.2237	**0.0063**	**0.0004**	-	**0.8986**	**0.9000**	**0.9416**	**0.8757**	**0.8135**
IL-2	0.1161	**0.0031**	0.1740	**0.0126**	0.3738	0.1567	0.3940	0.5906	0.2722	0.1316	0.4137	**0.0385**	**0.0000**	**0.0001**	**0.0010**	-	**0.9571**	**0.9692**	**0.9307**	**0.9465**
IL-4	0.1128	**0.0071**	0.2389	**0.0065**	0.1550	0.0550	0.1875	0.7758	0.5962	**0.0179**	0.3528	0.1100	**0.0000**	**0.0000**	**0.0009**	**0.0001**	-	**0.9272**	**0.9757**	**0.9610**
IL-5	0.1104	**0.0097**	0.2765	**0.0345**	0.3447	0.1487	0.3402	0.8246	0.1356	0.1694	0.6945	0.0588	**0.0008**	**0.0005**	**0.0001**	**0.0000**	**0.0003**	-	**0.8771**	**0.8963**
CXCL-1	0.1168	**0.0042**	0.1379	**0.0013**	0.1719	0.0708	0.2078	0.7437	0.6253	**0.0271**	0.3190	0.0789	**0.0001**	**0.0002**	**0.0020**	**0.0003**	**0.0000**	**0.0019**	-	**0.9614**
TNF-α	0.0815	**0.0002**	0.1611	**0.0034**	0.0623	**0.0196**	0.0851	0.9817	0.6442	**0.0045**	0.4956	**0.0285**	**0.0000**	**0.0007**	**0.0076**	**0.0001**	**0.0000**	**0.0011**	**0.0000**	-

P / r values[1]	Ear thickness	Clinical score	Histolog. score	Serum IgE	%CD3	%CD8/CD3	%CD19	%NK	%NKT	%CD4	%CD103/CD4	IFN-γ	IL-10	IL-12p70	IL-1ß	IL-2	IL-4	IL-5	CXCL-1	TNF-α
Ear thickness	-	0.5714	-0.1451	-0.4120	-0.7740	-0.2647	0.7503	0.4193	0.3614	-0.8131	0.8294	0.2773	**0.8518**	0.7677	0.7312	0.7740	0.5950	0.3578	0.6585	0.7209
Clinical score	0.0844	-	0.4762	-0.4864	-0.4563	0.1437	0.4694	0.0825	0.0107	-0.1825	0.4083	0.4020	0.5710	0.3831	0.3528	0.3856	0.1798	0.3044	0.4590	0.1622
Histolog. score	0.7095	0.0852	-	0.0358	-0.3579	-0.2085	0.3497	-0.2282	-0.6137	0.1966	-0.2194	0.2677	0.1497	-0.1699	-0.2552	-0.1463	-0.1376	0.1303	0.0510	-0.2021
Serum IgE	0.3584	0.1540	0.9218	-	0.3030	-0.7381	-0.2435	-0.6923	-0.8731	0.1599	-0.5351	-0.4174	-0.2301	-0.2652	-0.5261	-0.0412	0.0133	0.0083	-0.0103	-0.0777
%CD3	0.1245	0.2558	0.4305	0.6970	-	0.2873	**-0.9972**	-0.2929	0.1959	**0.7125**	**-0.7391**	-0.5196	**-0.9997**	-0.7499	-0.6401	-0.8779	-0.7384	-0.6778	-0.8574	-0.7767
%CD8/CD3	0.6669	0.7342	0.6537	0.2619	0.4903	-	-0.2702	0.0947	0.5094	0.0147	0.2005	0.8978	0.8399	**0.9817**	0.9389	**0.9756**	**0.9632**	0.8556	**0.9714**	**0.9843**
%CD19	0.1440	0.2406	0.4419	0.7565	**0.0000**	0.5175	-	0.2442	-0.2264	-0.7031	**0.7436**	0.4032	**0.9823**	0.6500	0.5219	0.7930	0.6280	0.5459	0.7660	0.6786
%NK	0.4822	0.8460	0.6226	0.3077	0.4815	0.8235	0.5600	-	0.6658	-0.5180	0.4680	0.8465	0.0544	0.6799	0.7890	0.5141	0.7034	0.7112	0.5483	0.6537
%NKT	0.5500	0.9800	0.1427	0.1269	0.6421	0.1973	0.5898	0.0715	-	-0.2096	0.3514	**0.9969**	0.4586	**0.9274**	0.9676	0.8089	0.9210	0.8374	0.8250	0.9095
%CD4	0.0942	0.6654	0.6726	0.8401	**0.0473**	0.9724	0.0517	0.1885	0.6184	-	**-0.8962**	-0.6246	-0.7111	-0.7551	-0.7526	-0.8345	-0.8124	-0.9364	-0.8488	-0.7769
%CD103/CD4	0.0824	0.3152	0.6365	0.4649	**0.0362**	0.6340	**0.0344**	0.2422	0.3933	**0.0026**	-	0.7802	0.9407	0.9323	0.8685	**0.9897**	0.9280	0.8691	**0.9824**	0.9473
IFN-γ	0.5948	0.2835	0.4861	0.2637	0.4804	0.1022	0.5968	0.1535	**0.0031**	0.3754	0.2198	-	0.2437	0.6406	**0.6969**	**0.6996**	**0.7077**	**0.8477**	**0.7776**	0.6347
IL-10	**0.0313**	0.1083	0.7006	0.5515	**0.0003**	0.1601	**0.0177**	0.9456	0.5414	0.2889	0.0593	0.5275	-	**0.7128**	0.5374	0.5468	0.5168	0.2428	0.5606	0.6544
IL-12p70	0.0747	0.3088	0.6622	0.4903	0.2501	**0.0183**	0.3500	0.3201	0.0726	0.2449	0.0677	0.0631	**0.0311**	-	**0.9036**	**0.8811**	**0.9024**	**0.6848**	**0.8721**	**0.9084**
IL-1ß	0.0987	0.3518	0.5075	0.1457	0.3599	0.0611	0.4781	0.2110	**0.0324**	0.2474	0.1315	**0.0370**	0.1356	**0.0008**	-	**0.7967**	**0.7594**	0.6536	**0.7429**	**0.7781**
IL-2	0.0708	0.3054	0.7073	0.9162	0.1221	**0.0244**	0.2070	0.4859	0.1911	0.1655	**0.0103**	**0.0359**	0.1277	**0.0017**	**0.0102**	-	**0.9414**	**0.8710**	**0.9608**	**0.8816**
IL-4	0.2129	0.6435	0.7241	0.9729	0.2616	**0.0368**	0.3720	0.2966	0.0790	0.1876	0.0720	**0.0329**	0.1543	**0.0009**	**0.0176**	**0.0002**	-	**0.8481**	**0.9388**	**0.9481**
IL-5	0.4862	0.4258	0.7384	0.9831	0.3222	0.1444	0.4541	0.2888	0.1626	0.0636	0.1309	**0.0039**	0.5290	**0.0418**	0.0562	**0.0022**	**0.0039**	-	**0.9128**	**0.6984**
CXCL-1	0.1550	0.2139	0.8963	0.9789	0.1426	**0.0286**	0.2340	0.4517	0.1750	0.1512	**0.0176**	**0.0136**	0.1164	**0.0022**	**0.0218**	**0.0000**	**0.0002**	**0.0006**	-	**0.8645**
TNF-α	0.1059	0.6768	0.6020	0.8424	0.2233	**0.0157**	0.3214	0.3463	0.0905	0.2231	0.0527	0.0663	0.0558	**0.0007**	**0.0135**	**0.0017**	**0.0001**	**0.0364**	**0.0026**	-

[1]Clinical and histological parameters were correlated with immunological parameters and P (black and red) and r (grey) values are given in the tables for the control group (upper panel) and for the XOS group (lower panel). Significant correlations are highlighted in bold and colored red.
